# Assessment of T Regulatory Cells and Expanded Profiling of Autoantibodies May Offer Novel Biomarkers for the Clinical Management of Systemic Sclerosis and Undifferentiated Connective Tissue Disease

**DOI:** 10.1155/2013/390563

**Published:** 2013-05-29

**Authors:** Paola Cordiali-Fei, Anna Mussi, Giovanna D'Agosto, Elisabetta Trento, Valentina Bordignon, Silvana Trincone, Antonella Vento, Isabella Sperduti, Antonio Cristaudo, Fabrizio Ensoli

**Affiliations:** ^1^Clinical Pathology and Microbiology, San Gallicano Dermatology Institute, Via Elio Chianesi 53, 00144 Rome, Italy; ^2^Clinical Dermatology, San Gallicano Dermatology Institute, Via Elio Chianesi 53, 00144 Rome, Italy; ^3^Biostatistic Unit, Regina Elena Cancer Institute, Via Elio Chianesi 53, 00144 Rome, Italy

## Abstract

In order to identify disease biomarkers for the clinical and therapeutic management of autoimmune diseases such as systemic sclerosis (SSc) and undifferentiated connective tissue disease (UCTD), we have explored the setting of peripheral T regulatory (T reg) cells and assessed an expanded profile of autoantibodies in patients with SSc, including either limited (lcSSc) or diffuse (dcSSc) disease, and in patients presenting with clinical signs and symptoms of UCTD. A large panel of serum antibodies directed towards nuclear, nucleolar, and cytoplasmic antigens, including well-recognized molecules as well as less frequently tested antigens, was assessed in order to determine whether different antibody profiles might be associated with distinct clinical settings. Beside the well-recognized association between lcSSc and anti-centromeric or dcSSC and anti-topoisomerase-I antibodies, we found a significative association between dcSSc and anti-SRP or anti-PL-7/12 antibodies. In addition, two distinct groups emerged on the basis of anti-RNP or anti-PM-Scl 75/100 antibody production among UCTD patients. The levels of T reg cells were significantly lower in patients with SSc as compared to patients with UCTD or to healthy controls; in patients with lcSSc, T reg cells were inversely correlated to disease duration, suggesting that their levels may represent a marker of disease progression.

## 1. Introduction

Systemic sclerosis is an autoimmune systemic disease characterized by diffuse fibrosis and vasculopathy. The diffuse alteration of small blood vessel leads to tissue ischemia and fibroblast stimulation, which result in accumulation of collagen in the skin and internal organs [1]. Patients with SSc can be classified into distinct clinical categories, characterized by different outcomes and life expectancy [2]. According to the extent of skin involvement, patients are classified as limited cutaneous scleroderma (lcSSc) and diffuse cutaneous scleroderma (dcSSc) [3]. In lcSSc, fibrosis is mainly restricted to the hands, arms, and face. Raynaud's phenomenon is generally present for several years before fibrosis appears and pulmonary hypertension represents a frequent clinical complication. In dcSSc, which represents a rapidly progressing disease, a large area of the skin is affected by fibrosis which extends to one or more internal organs. Autoantibodies characteristically targeting nuclear antigens are recognised as one of the hallmarks of SSc and their presence is considered a key feature for the diagnosis. In addition, the presence of different types of antinuclear antibodies (ANAs) appears to be associated with distinct outcomes of the disease including clinical severity [4]. Although the current criteria of the American College of Rheumatology for SSc staging do not include the presence of ANAs [5, 6], their detection might offer an additional tool for the clinical management of the disease, since they might help distinguish patients with an early SSc from those presenting an undifferentiated connective tissue disease (UCTD). According to the more recently proposed criteria [7], UCTD is characterized by a persistent oligosymptomatic condition (at least 3 years) which might evolve into aggressive autoimmune diseases as SSc, systemic lupus erythematosus, primary Sjögren's syndrome, mixed connective tissue disease (MCTD), systemic vasculitis, polydermatomyositis (PM/DM), and rheumatoid arthritis (RA) [8]. The laboratory determination of the autoantibody profile represents a useful tool for both diagnosis and characterization of distinct clinical manifestations of autoimmune diseases; however, their presence or titer tends to persist during the course of the disease, even following therapeutic interventions [4]. Indeed, both in SSC and UCTD the role of autoantibodies in inducing the disease is, as yet, unclear [9]. However, some authors have reported a favorable outcome in SSc patients who lose anti-topo I antibody during the disease course [10], and previous studies have shown a marked reduction of organ inflammation following the suppression of autoantibody production both in human [11] and experimental lupus [12], strongly, though indirectly, suggesting that antibodies reacting with self-components can trigger a chronic, site-specific, inflammation, which, in turn, can be responsible for organ damage. In this view, accumulating evidence has pointed at the pivotal role played by T reg cells in autoimmune diseases, since these cells are key for the regulation, including the initiation as well as the termination, of the adaptive immune response [13]. Previous studies suggested that T reg cells may play a role either in controlling autoantibody production [14] or in limiting autoantibody-induced inflammation through IL-10 production [15, 16] or downregulation of costimulatory molecules on APCs [17]. In order to identify “disease biomarkers” useful for the clinical and therapeutic management of autoimmune disorders, in the present study we assessed an extended panel of nuclear, nucleolar, and cytoplasmic autoantigens, including those associated with SSc (Topoisomerase-I, Cenp-A/B, RNAP III, Th/To, Fibrillarin, PDGFR) as well as dermatomyositis (Mi-2, Jo-1, PL-7, PL-12, EJ, OJ, SRP) or other overlapping syndromes (PM-Scl 75 e PM Scl 100, Ku, Ro-52, NOR 90) [18] facing the determination of the regulatory T cell levels in patients with different clinical forms of SSc and in subjects presenting with a UCTD.

## 2. Patients and Methods

### 2.1. Study Design

A cross-sectional study was performed on consecutive patients with diagnosed or suspected SSc incoming the autoimmune outpatients' clinical department (San Gallicano Dermatology Institute) between January 2011 and December 2012. The diagnosis of cSSc was based on the presence of the Raynaud phenomenon associated to abnormal nailfold capillary examination and dermal skin thickness evaluated by clinical palpation of 17 areas of the body [19]. In limited disease (lcSSc) cutaneous involvement was distal to the elbows, knees, and clavicles; in diffuse disease (dcSSc) cutaneous involvement was present also in arms, chest, abdomen, back, or thighs according to the classification criteria established by Carwile LeRoy et al. [3, 20]. Blood samples were collected upon informed consent at the time of the first medical examination and consisted of 3 mL EDTA for flow cytometry analysis and 3 mL for serological assays.

### 2.2. Patients

The diagnosis of UCTD was based on clinical and serological manifestations suggesting a systemic connective autoimmune disease (arthralgias, Raynaud's disease, ANA reactivity different from antitopoisomerase I and anticentromere, disease duration) [7]. Forty-eight patients were examined in the study: 11 had dcSSc, 14 had lcSSc, and 23 were diagnosed as UCTD. Forty-five of them were women and 3 men. The median age and disease duration from diagnosis to blood sampling as well as a description of the clinical symptoms are summarized in Table 1. All patients were under therapy with 0,5–2 ng of iloprost/kg/min. The control group was composed by 15 healthcare workers, 12F/3 M, median age 53 yrs (range 36−64). All subjects were enrolled upon signature of written informed consent. 

### 2.3. Autoantibodies

An immunofluorescence assay (IFA) (Kallestad HEp-2 cell line substrate, Bio-Rad Laboratories, Redmond, WA) was used to detect SSc-associated autoantibodies. An IFA titre greater than 1 : 80 dilution was considered positive and the specific fluorescence pattern was recorded. Sera were further analyzed by the Bioplex 2200 ANA screen system (Bio-Rad Laboratories, Hercules, CA), a fully automated system based on multiplexed bead technology, which allows the simultaneous detection of different autoantibodies directed toward a panel of autoantigens including dsDNA, chromatin, centromere-B, Scl-70, RNP (68 kDa, RNP-A), SSA (52 and 68 kDa), SSB, Sm, Sm/RNP, P ribosomal, and Jo-1. The sera were further tested by two distinct immunoblot assays (Euroline Systemic Sclerosis Nucleoli profile and Euroline Myositis profile, Euroimmun, Germany, resp.) against purified (Scl-70) or recombinant nuclear (CENP A and CENP B, SSA 52, Ku, Mi-2), nucleolar (Th/To, RNAP III, Fibrillarin, NOR 90, PM-Scl 75, PM-Scl 100), and cytoplasmic antigens (Jo-1, SRP, PL-7, PL-12, EJ, OJ), following the manufacturer's instructions.

### 2.4. Immunophenotyping

Peripheral blood lymphocyte subsets were examined by flow cytometry. Absolute lymphocyte counts were determined by BD FACSCanto II flow cytometer using BD FACSCanto clinical software v2.4 (BD Biosciences) after incubation of 50 uL of whole blood with 20 uL of a mix of monoclonal antibodies (anti-CD45/CD3/CD4/CD8/CD19/CD16-CD56, BD Multitest 6-color TBNK reagent; BD Trucount tubes). T regulatory cells were identified through surface expression of CD4/CD25 antigens and intracellular expression of FoxP3 (Human T reg Flow Kit, Biolegend, San Diego, CA).

### 2.5. Statistical Analysis

The results (i.e., different responses toward distinct antigens) were compared by contingency tables and *X* ^2^ values using the GraphPad Prism5 software (GraphPad Software, Inc., San Diego, CA, USA). The relationship between clinical parameters and serological profiles was assessed by Multiple Correspondence Analysis of data using the SPSS software (SPSS Statistics for Windows, Version 19.0. IBM Corp. Armonk, NY). Results of lymphocyte subsets, including T regulatory cells, were compared among the different subject groups by nonparametric test using the GraphPad Prism5 software (GraphPad Software, Inc., San Diego, CA, USA).

## 3. Results

### 3.1. Autoantibody Profiles

**Figure 1:**
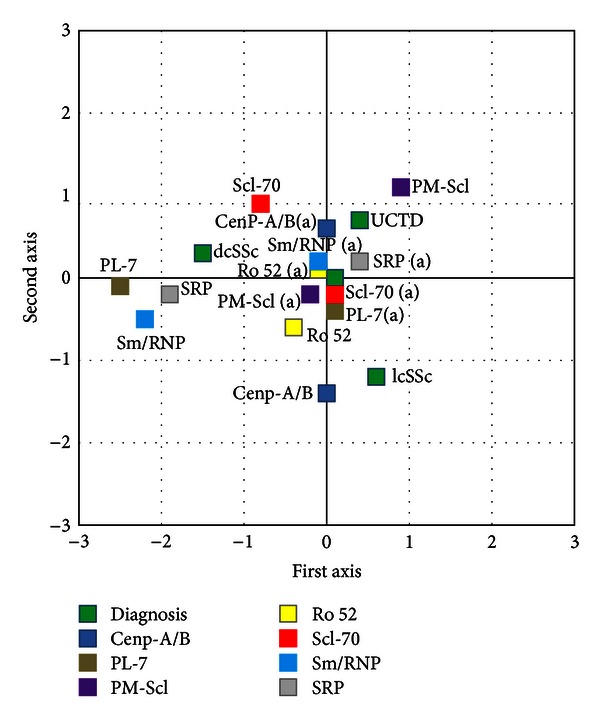
Multiple correspondence analysis showing the association between clinical and serological variables.

The frequency of antinuclear antibodies and the distribution of the different fluorescence patterns are shown in Table 2. All patients with lcSSc had anti-nuclear antibodies as determined by IFA. These were mainly directed against centromeric antigens (78,6%). Patients with dcSSc had antibodies against both nuclear and nucleolar antigens in 54,5% of cases while UCTD patients had speckled antinuclear (65,2%) or antinucleolar (21.7%) responses. The antibody profiles determined by Bioplex and immunoblot are summarized in Table 3. None of the collected sera had antibodies against fibrillarin, OJ, or EJ as well as Th/To antigens. As expected, a highly significant association was found between production of anti-Cenp A/B antibodies and lcSSc as well as between anti Scl-70 and dcSSc. A significantly high frequency of patients with dcSSc had also antibodies against PL-7/12 or SRP antigens (27.3% and 36.4%, resp.). Among UCTD patients two subgroups could be distinguished on the basis of anti-PM-Scl (26.0%) or anti-RNP (30.4%) antibodies. Multiple correspondence analysis (MCA), a descriptive and exploratory technique designed to analyze simple two-way and multiway data, was used to evaluate the possible association among the production of specific antibodies and the clinical outcome. These data, which are shown in Figure 1, further confirmed the recognized association between lcSSc and anti-Cenp A/B as well as between dcSSc and anti-Scl70 antibodies. Interestingly, anti-PM/Scl responses were mainly associated to patients with UCTD.

### 3.2. Lymphocyte Subsets

**Figure 2:**
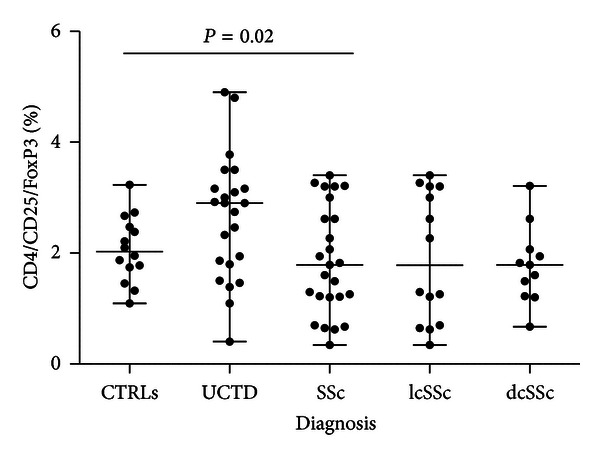
Levels of T reg cells (CD4+/CD25+/FoxP3+) in controls and patients with UCTD or cSSc are significantly different.

**Figure 3:**
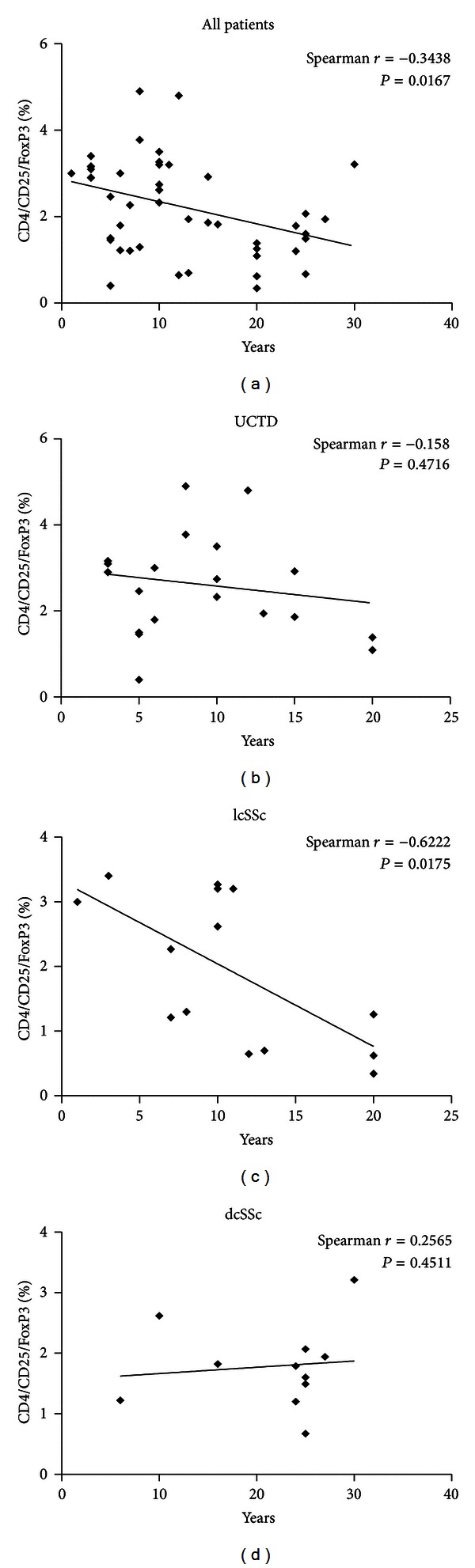
Correlation of T reg cell levels and disease duration in all patients (a), patients with UCTD (b), patients with SSc, limited (c), or diffuse (d) disease.

The analysis of lymphocyte subsets is described in Table 4. The results showed that patients with SSc, either affected by lcSSc or dcSSc, have higher T cell counts and significantly lower frequencies of T reg cells (also depicted in Figure 2) as compared to UCTD patients or to healthy controls. To further explore the setting of T reg cells during the progression of the disease, the relationship between T reg cell levels and disease duration was examined (Figure 3). The results showed a significant correlation between disease duration and reduced T reg cell percentages (Figure 3(a)), although a strong statistical significance was found only in the group of patients with lcSSc (Figure 3(c)).

## 4. Discussion and Conclusions

In the present paper,we have assessed an expanded profile of autoantibodies and determined the levels of peripheral T reg cells in patients with SSc, including either limited or diffuse disease, and in patients presenting with clinical signs and symptoms of UCTD, in order to identify disease biomarkers useful for the clinical and therapeutic management. The results indicate that testing an extended antibody profile may provide possible advantages for the clinical classification of patients with SSc and UCTD, while assessing the levels of T reg lymphocytes might help monitoring disease progression. In fact, a multiparametric profiling of antibodies directed towards nuclear, nucleolar, and cytoplasmic antigens, including well-recognized molecules as well as less frequently studied antigens helped at identifying distinctively different clinical outcomes. The use of an extended panel of antigens, mainly related to PM or DM, has been previously proposed in order to identify SSc patients with overlapping syndromes [21]. None of our patients had clinical evidence of an overlapping syndrome (i.e., PM/SSc). However, anti-SRP and anti-PL7/12 antibodies proved useful to identify the most severe clinical forms of SSc, which, in turn, presented with the lowest levels of T reg cells. As previously described from different authors, we also found an association between anticentromeric antigens and lcSSc or antitopoisomerase-I and dcSSc [21]. In fact, according to the analysis of results from EULAR study, ACA and antitopoisomerasi are independent predictors of disease presentation/organ involvement [22]. However, in this latter clinical setting, corresponding to the most severe of the autoimmune disorders included in this study, we found also a significatively higher prevalence of antisignal recognition particle (SRP) and/or anti-PL-7/12 antibodies as compared to the lcSSc or the UCTD syndromes. SRP is a ribonuclear protein that regulates protein traslocation across the endoplasmic reticulum membrane during protein synthesis. Anti-SRP antibodies were initially found in patients with PM [20], and they have been subsequently recognized as myositis-specific antibodies [23]. However, the presence of anti-SRP antibodies has been occasionally reported in patients with other immunologic disorders, including SSc, however in the absence of an evident myopathy [24]. Nevertheless, skeletal muscle involvement represents a well-recognized characteristic of SSc [25–28]. Anti-PL-7, threonyl-tRNA synthetase, and anti-PL-12, alanyl-tRNA synthetase, antibodies have been previously implicated in the pathogenesis of the antisynthetase syndrome, a disease characterized by varying degrees of interstitial lung disease, myositis, arthropathy, fever, Raynaud's phenomenon, and mechanic's hands [29]. A recent analysis of the clinical profile of patients with connective tissue disease, anti-PL-12 autoantibodies suggest a strong association with idiopathic lung fibrosis rather than myositis or arthritis [30]. In the group of UCTD patients examined in the present study, two main autoantibodies were present: anti-RNP or anti-PM/Scl antibodies. Anti-RNP have been associated with the mixed connective tissue disease (MCTD) while anti-PM-Scl antibodies have been described in SSc patients with milder symptoms, eventually susceptible to evolve to SSc within 5–15 years from the onset of clinical symptoms [31]. However, these patients presented a stable clinical outcome characterized by the presence of typical RP/SSc-like capillaroscopic findings during a period of several years of followup. A clinical and laboratory followup over a more extended period could be necessary to establish or exclude the eventual evolution towards a definite connective disease. 

The assessment of T reg lymphocytes may represent an important parameter to measure the immune dysregulation underlying autoimmune disorders. In fact, by either initiating or terminating the adaptive immune response [13], T reg cells may play a central role in the pathogenesis of autoimmunity. It has been shown that an increased expression of genes associated with SSc susceptibility and/or disease manifestations plays a major role also in the regulation of the immune system [32]. Furthermore, an inadequate number of T reg lymphocytes can lead to autoimmunity in humans, as it is clearly shown in patients with immune dysregulation, polyendocrinopathy, enteropathy, and X-linked (IPEX) syndrome, who completely lack T reg cells as results of a mutation in FoxP3 [33]. Indeed, recent studies aimed at assessing the number of T reg cells, pointed at a decrease of T reg frequencies in individuals with immunologic disorders such as rheumatic diseases, including scleroderma [34–36]. However, authors which previously afforded the analysis of T cell reg in SSc patients, underlined an alteration of T cell homeostasis in these patients with an increase of peripheral CD4 T cells and of T reg cells, correlated to disease severity [37, 38] or irrespective of disease phenotype but associated to an impaired regulatory function [39]. In the present study, the results of the analysis of lymphocyte subsets confirmed an increase of CD4 T cells, but in association with a decrease of the frequency of CD4 T reg lymphocytes in SSc patients. In fact, decreased percentages of T reg lymphocytes were found either in the limited or the diffuse disease, although in the dcSSc, which arise as severe clinical condition, their levels do not vary with disease duration, and in the lcSSc, which can be milder at the onset but advance with time, T reg levels decrease according to disease duration, suggesting a relationship between the setting of T reg cells and the progression of the disease. Of note, these results did not depend upon therapeutic intervention since all the patients examined in this study underwent the same therapeutic protocol (iloprost 0,5–2,0 ng/kg/min). This notion also suggests that such therapeutic intervention, though effective at ameliorating some clinical signs and symptoms, exerts a limited impact on regulatory immune circuitries, which appears to play a key pathogenic role and to be chronically and progressively altered in autoimmune disorders. This further emphasizes the need of novel therapeutic strategies capable of targeting regulatory cells/signals involved in the generation and termination of the adaptive immune response. In fact, although numerous studies have focused on the pathogenic mechanism of immune activation and tissue fibrosis in SSc [40] and potential therapeutic targets have been identified [41], efficacious treatment strategy for this disease has not been established yet.

Taken together, our data indicate the following: (i) dcSSc, which corresponds to a severe autoimmune disorder and is associated to the presence of several class of autoantibodies, presents with very low levels of T regs, which do not appear to depend upon disease duration; (ii) lcSSc, which is characterized by high titers of anti-centromere antibodies from the earlier clinical stages and generally starts with mild symptoms which progress during time, presents levels of T reg which are progressively lower in association with disease duration; (iii) UCTD, which is characterized by an undifferentiated onset, which could remain clinically undetermined for an indefinite time or may even show a clinical remission [8], presents with a reduced spectrum and low levels of autoantibodies and shows levels of T reg which are comparable to healthy controls. These observations support the notion that T reg lymphocytes may play a central role in the pathogenesis of SSc and that the determination of the levels of peripheral T reg cells may represent a useful tool in the diagnosis, prognosis, and monitoring of patients presenting with clinical signs and symptoms of autoimmune inflammatory diseases and might prove a key disease biomarker for the clinical and therapeutic management of major autoimmune disorders.

## Figures and Tables

**Table 1:** Clinical and demographic characteristics of patients.

Sex F/M	lcSSc (14)	dcSSc (11)	UCTD (23)
	14/0	8/3	23/0
Age range (median yrs)	41–66 (56)	35–80 (68)	18–73 (51)
Disease duration range (median yrs)	7–20 (12)	6–30 (24)	3–20 (8)
Raynaud's phenomenon P/N	14/0	11/0	23/0
Fingertip ulcers P/N	14/0	11/0	14/9
Pulmonary arterial hypertension P/N	0/14	7/4	2/21
Pulmunary fibrosis P/N	0/14	7/4	0/23
Therapy (iloprost)	Yes	Yes	Yes

**Table 2:** Frequency of anti-nuclear antibodies and fluoroscopic patterns.

Anti-nuclear antibodies	Diagnosis			*P*
(ANA)	lcSSc (14)	dcSSc (11)	UCTD (23)	
P/N	14 (100%)	8 (73%)	20 (87%)	
Titer (range)	1 : 5120 (1 : 640–1 : 5120)	1 : 5120 (1 : 80–1 : 5120)	1 : 320 (1 : 80–1 : 1280)	
Centromere	**11 (78.6**%**) **	1 (12.5%)	0	<0.0001
Nucleolar	3 (21.4%)	0	5 (25.0%)	n.s.
Homogeneous and nucleolar	0	**6 (75.0**%**) **	0	<0.0001
Speckled	0	0	**15 (75.0**%**) **	<0.0001
Mitotic Spindle	0	1 (12.5%)	0	n.s.

**Table 3:** Frequency of reactivity to nuclear, nucleolar, or cytoplasmic antigens.

Antibody reactivity	Diagnosis			*P*
	lcSSc (14)	dcSSc (11)	UCTD (23)	
	P/N	P/N	P/N	

ANA (I.F.A.)	14/0	11/0	18/5	0.02
Cenp-A/B	11/3	3/8	0/23	<0.0001
Scl-70	0/14	6/5	0/23	<0.0001
Ro-52	2/12	3/8	1/22	
RNP	0/14	0/11	7/16	0.011
Mi-2	1/13	1/10	0/23	
PM/Scl	1/13	1/10	5/18	
NOR	2/12	1/10	2/21	
Ku	1/13	2/9	3/20	
RP	1/13	0/11	1/22	
PL-7-12	0/14	3/8	0/23	0.005
SRP	0/14	4/7	1/22	0.005
Jo-1	0/14	0/11	0/23	
≥2 antigens	4/7	8/6	3/20	0.017

**Table 4:** Peripheral lymphocyte subsets including T reg cells in patients and controls.

Lymphocyte subsets							
Diagnosis	CD3+ n/mmc median value range	CD3+CD4+ n/mmc median value range	CD3+CD8+ n/mmc median value range	CD19+ n/mmc median value range	CD16+CD56+ n/mmc median value range	CD4/CD8 ratio median value range	CD4/CD25/FoxP3 % of CD4+ cells median value range
lcSSc	1538* 1004–2666	1196* 683–1939	351 221–727	250 99–763	162 54–475	3.05* 1.88–4.53	1.67* 1.21–3.27
dcSSc	1704* 952–3426	1317* 592–2637	506 170–938	159 45–1031	158 90–752	2.99 1.11–7.1	1.85* 1.22–3.21
UCTD	1308 490–2506	967 282–1830	437 143–694	181 36–866	186 25–397	2.30 0.56–5.11	2.90 0.4–4.90
CTRLS	1160 1300–1680	840 738–1040	504 272–760	231 100–310	294 100–810	2.10 1.2–2.9	2.10 1.1–3.2
*P*	<0.0001	0.0003	0.0800	0.0440	0.0860	0.0007	0.0030
